# Effects of 3‐HAA on HCC by Regulating the Heterogeneous Macrophages—A scRNA‐Seq Analysis

**DOI:** 10.1002/advs.202207074

**Published:** 2023-04-04

**Authors:** Chen Xue, Xinyu Gu, Qiuxian Zheng, Qingmiao Shi, Xin Yuan, Qingfei Chu, Junjun Jia, Yuanshuai Su, Zhengyi Bao, Juan Lu, Lanjuan Li

**Affiliations:** ^1^ State Key Laboratory for Diagnosis and Treatment of Infectious Diseases National Clinical Research Center for Infectious Diseases National Medical Center for Infectious Diseases Collaborative Innovation Center for Diagnosis and Treatment of Infectious Diseases The First Affiliated Hospital Zhejiang University School of Medicine Hangzhou 310003 China; ^2^ Division of Hepatobiliary and Pancreatic Surgery Department of Surgery The First Affiliated Hospital Zhejiang University School of Medicine Hangzhou 310003 China

**Keywords:** 3‐hydroxyanthronic acid, cytometry by time‐of‐flight, hepatocellular carcinoma, macrophage, single‐cell RNA sequencing

## Abstract

Kynurenine derivative 3‐hydroxyanthranilic acid (3‐HAA) is known to regulate the immune system and exhibit anti‐inflammatory activity by inhibiting T‐cell cytokine secretion and influencing macrophage activity. However, the definite role of 3‐HAA in the immunomodulation of hepatocellular carcinoma (HCC) is largely unexplored. An orthotopic HCC model and treated with 3‐HAA by intraperitoneal injection is developed. Furthermore, cytometry by time‐of‐flight (CyTOF) and single‐cell RNA sequencing (scRNA‐seq) analyses are carried out to define the immune landscape of HCC. It is found that 3‐HAA treatment can significantly suppress tumor growth in the HCC model and alter the level of various cytokines in plasma. CyTOF data shows that 3‐HAA significantly increases the percentage of F4/80^hi^CX3CR1^lo^Ki67^lo^MHCII^hi^ macrophages and decreases the percentage of F4/80^lo^CD64^+^PD‐L1^lo^ macrophages. scRNA‐seq analyses demonstrate that 3‐HAA treatment is proved to regulate the function of M1 macrophages, M2 macrophages, and proliferating macrophages. Notably, 3‐HAA inhibits the proinflammatory factors TNF and IL‐6 in multiple cell subsets, including resident macrophages, proliferating macrophages, and pDCs. This study reveals the landscape of immune cell subsets in HCC in response to 3‐HAA, indicating that 3‐HAA may be a promising therapeutic target for HCC.

## Graphical abstract

Multiple‐cell subsets, such as resident macrophages, proliferating macrophages, and pDCs, display significant inhibition of the proinflammatory factors TNF and IL‐6 by 3‐HAA. CXCL13 is enriched in kupffer cells and upregulated in the 3‐HAA‐treated group. 3‐HAA inhibits CSF1 and CSF2 in M2 macrophages. 3‐HAA may be involved in remodeling the tumor immune microenvironment and provides novel treatment strategies for HCC.

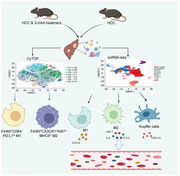

## 1 Introduction

Primary liver cancer was the sixth most prevalent cancer and the fourth leading cause of cancer‐related mortality globally.^[^ ^1^ ^]^ Hepatocellular carcinoma (HCC) is the most common type of primary liver cancer, accounting for 75%–85% of cases. Infection with the hepatitis B and C viruses is a prominent risk factor for the onset and progression of HCC.^[^ ^2^ ^]^ Although various treatment options based on liver resection, locoregional therapies, targeted therapy, chemotherapy, and liver transplantation are available, many questions remain unanswered. Only liver transplantation is considered an ideal cure, which is constrained by the availability of donor grafts.^[^ ^3^, ^4^ ^]^ Considering the aforementioned factors, advancements in HCC treatment are still required.

Tryptophan (Trp) is an essential amino acid in humans that is involved in protein synthesis and serves as a precursor for the synthesis of many microorganisms and host metabolites. Notably, Trp is an important energy source for the regulation of immune cell function.^[^ ^5^ ^]^ Disorders of Trp metabolism can lead to apoptosis and dysfunction of immune cells, which is conducive to the formation of an immunosuppressive microenvironment, thereby affecting the efficacy of immunotherapy for HCC.

There are three pathways involved in Trp metabolism, with the kynurenine (Kyn) metabolism pathway accounting for 95% of Trp metabolism. Kyn is the primary product of the Trp metabolism pathway, which is catalyzed by tryptophan 2,3‐dioxygenase and indoleamine 2,3‐dioxygenase.^[^ ^6^ ^]^ Kyn is converted into 3‐hydroxyanthranilic acid (3‐HAA), 3‐hydroxykynurenine, and other metabolites via kynurenine 3‐monooxygenase, kynurenine aminotransferases, and kynureninase.^[^ ^7^ ^]^ Studies have reported that the Kyn derivative 3‐HAA suppresses tumor growth and sensitizes HCC to sorafenib.^[^ ^8^, ^9^ ^]^ In addition, 3‐HAA is known to regulate the immune system and exhibits anti‐inflammatory activity by inhibiting T‐cell cytokine secretion and influencing macrophage activity.^[^ ^10^, ^11^ ^]^ However, the precise role of 3‐HAA in cancer immunity is largely unknown.

This study aimed to elucidate the effects and immunoregulation of 3‐HAA on HCC. The levels of Kyn derivative 3‐HAA in HCC tissues and adjacent nontumor tissues were determined using liquid chromatography–mass spectrometry (LC–MS). We demonstrated that 3‐HAA treatment can significantly suppress tumor growth in the HCC model. Furthermore, cytometry by time‐of‐flight (CyTOF) and single‐cell RNA sequencing (scRNA‐seq) analyses were carried out to define the immune cell landscape of HCC. Not only did 3‐HAA increase the frequency of F4/80^hi^CX3CR1^lo^Ki67^lo^MHCII^hi^ macrophages and decreased the percentage of F4/80^lo^CD64^+^PD‐L1^lo^ macrophages, but also it regulated the function of M1 macrophages, M2 macrophages, and proliferating macrophages. Our findings can precisely define the immune cell subsets that are relevant to HCC, and 3‐HAA treatment could be a promising treatment strategy for HCC.

## 2 Results

### 2.1 The Level of 3‐HAA was Lower in Tumor Tissues than in Adjacent Nontumor Tissues of HCC Patients

**Figure 1:**
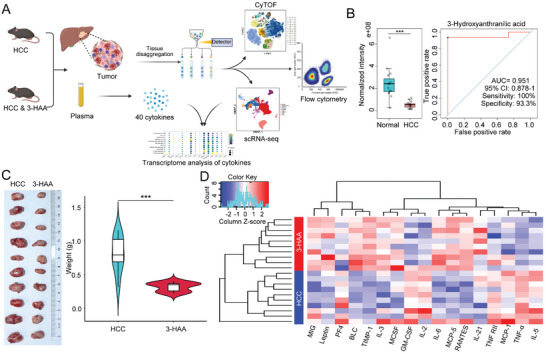
Overview of workflow and the anti‐tumor effects of 3‐HAA in HCC. A) The workflow of our study. B) LC‐MS examined the level of the metabolite 3‐HAA in tumor tissues compared with adjacent nontumor tissues in HCC patients (*n* = 15); Time‐dependent ROC curve for 3‐HAA in HCC. C) 3‐HAA treatment can significantly inhibit the growth of the tumor in the mice model of HCC. D) The different cytokines between the HCC and 3‐HAA groups. Red: high expression; Blue: low expression. ***P < 0.001. HCC, hepatocellular carcinoma; 3‐HAA, 3‐hydroxyanthranilic acid; LC‐MS, liquid chromatography–mass spectrometry; CyTOF, cytometry by time‐of‐flight; scRNA‐seq, single‐cell RNA sequencing.

The analytical procedure describing our study is shown in **Figure** **1**A. In a previous study, we found that the metabolic patterns (such as abnormal tryptophan metabolism) of HCC tissues were distinct from those of adjacent nontumor tissues.^[^ ^12^ ^]^ Here, we performed LC–MS to explore the different metabolites between 15 pairs of tumor tissues and adjacent nontumor tissues of HCC patients. It revealed that the level of the Kyn derivative 3‐HAA was lower in tumor tissues than in adjacent nontumor tissues, and the time‐dependent receiver operating characteristic (ROC) analyses for the survival prediction of 3‐HAA yielded the area under the curve of 0.951 (Figure 1B), suggesting that the level of 3‐HAA could be associated with the development of HCC.

### 2.2 3‐HAA Treatment Inhibits HCC Growth in the Mice Model of HCC

To further identify the role of 3‐HAA in the onset and progression of HCC, we developed an orthotopic HCC model and found that 3‐HAA treatment can significantly inhibit the growth of tumor in mice. Both tumor weights and volumes were significantly reduced in the 3‐HAA‐treated group (3‐HAA group) compared to the control group (HCC group) (Figure 1C). To further investigate the potential role of 3‐HAA in regulating the immune microenvironment, a total of 40 cytokines were analyzed in mice plasma using the Quantibody Mouse Inflammation Array Kit. The results showed that TNF‐*α*, TNF‐RII, IL‐5, and other pro‐inflammatory factors reduced significantly in 3‐HAA group. In addition, we observed that MCP‐1 was significantly elevated in the HCC group, suggesting increased local infiltration of chemotactic and activated macrophage (Figure 1D). The results indicating that 3‐HAA may modulate the anti‐tumor immune response by regulating the secretion of cytokines.

### 2.3 Immunological Characterization by CyTOF

**Figure 2:**
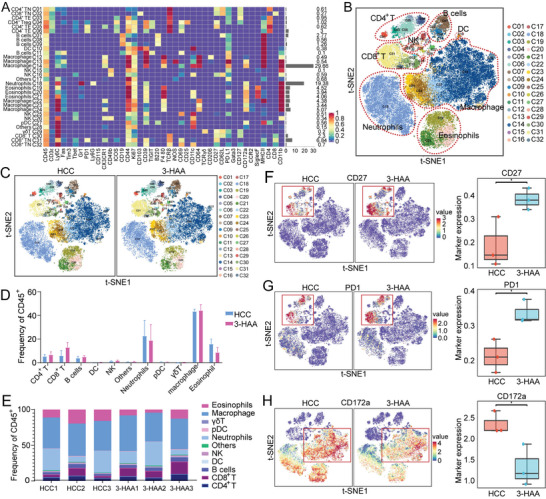
CyTOF analysis of intratumoral immune cells in HCC mice. A) The heatmap showing the expression of all selected panel markers for 32 cell clusters; Red: high expression; Purple: low expression. B) The t‐SNE plot presenting CyTOF data from all groups of immune cells in tumor tissue. C) The t‐SNE plot presenting CyTOF data from HCC group and 3‐HAA group of immune cells in tumor tissue. D) The proportions of distinct cell subsets (*n* = 3 per group). E) The proportional distribution of the distinct immune cell subtypes. F‐H) t‐SNE diagram and statistical analysis of the difference between two groups of cell functional markers CD27, PD‐1, and CD172. *P < 0.05. HCC, hepatocellular carcinoma; 3‐HAA, 3‐hydroxyanthranilic acid; t‐SNE, *t*‐distributed stochastic neighbor embedding.

To better understand the effect of 3‐HAA treatment on the tumor immune landscape, we collected tumor tissues from HCC mice and confidently defined immune cells for characterization using CyTOF analysis. We collected the immune cells from all samples and selected all surface and functional markers from the project panel for cluster analysis. We identified 32 distinct clusters (**Figure** **2**A; Table S1, Supporting Information). In addition, t‐distributed stochastic neighbor embedding (t‐SNE) plots were applied to display the expression level of 40 different markers in 32 clusters, which were analyzed using the PhenoGraph algorithm (Figure S1). Positional clustering of immune cell subpopulations was observed in t‐SNE plots, as expected: CD8^+^ T cells, CD4^+^ T cells, B cells, dendritic cells (DCs), natural killer (NK) cells, neutrophils, plasmacytoid dendritic cells (pDCs), gamma‐delta T (*γδ*T) cells, macrophages, and eosinophils (Figure 2B,C). Although there was no significant difference in the number of large cell subset between the 3‐HAA group and HCC group (Figure 2D,E), we found that functional markers CD27 and PD1 were increased in the 3‐HAA group compared to the HCC group, suggesting immune cells were functionally activated after 3‐HAA intervention (Figure 2F,G). CD172a is mainly expressed in monocytes/macrophages, which is an inhibitory immune receptor by interacting with the ligand CD47 to inhibit the phagocytosis of immune cells. The expression of this marker decreased in the 3‐HAA group, indicating that the phagocytosis function of macrophages was improved after 3‐HAA treatment (Figure 2H). These findings suggest that 3‐HAA may affect HCC progression by regulating immune cell function.

### 2.4 3‐HAA Regulates the Heterogeneity of Tumor‐Associated Myeloid Cells (TAMCs) in the Mice Model of HCC

**Figure 3:**
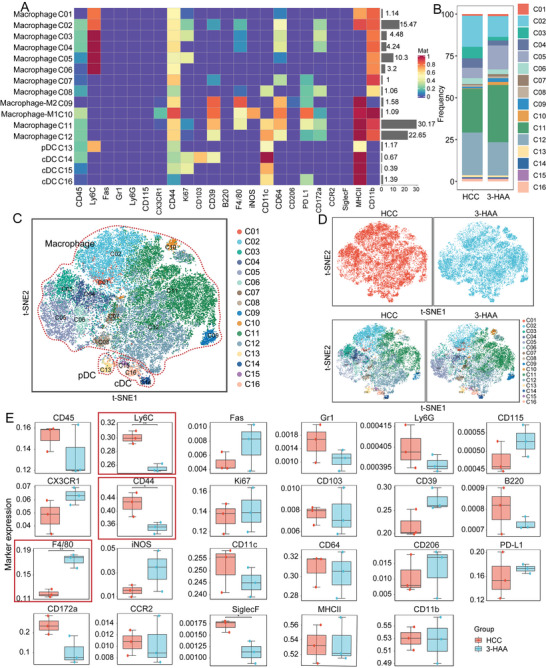
CyTOF analysis of TAMCs in HCC mice. A) Heatmaps of the expression levels of myeloid cell‐surface markers among myeloid cell subpopulations. Red: high expression; Purple: low expression. B) The proportional distribution of the myeloid cell subpopulations. C and D) t‐SNE plot of all groups and each indicated group of TAMCs populations. E) Bar plots of frequency and distribution for selected expression markers. *P < 0.05; **P < 0.01. HCC, hepatocellular carcinoma; 3‐HAA, 3‐hydroxyanthranilic acid; t‐SNE, t‐distributed stochastic neighbor embedding.

TAMCs are important cellular components of solid tumors, which include many different cell subtypes such as monocytes, macrophages, and dendritic cells. Notably, TAMCs are highly plastic and can divide into a variety of phenotypes with functions ranging from immunosuppression to immunostimulatory.^[^ ^13^ ^]^ Therefore, we further investigate the changes in TAMCs using CyTOF analysis (**Figure** **3**A,B; Table S2, Supporting Information). The t‐SNE map of TAMCs clustering revealed a significant difference between the 3‐HAA group and HCC group (Figure 3C,D). The expression of immune markers in the integrated data was visualized using t‐SNE (Figure S2, Supporting Information). F4/80 was a surface‐specific marker of macrophage, CD44 is positively correlated with grade of malignancy and is negatively related to prognosis in various types of tumors, CD44^+^ TAMCs are in M2 phenotype, suggesting the frequency of M2 phenotype is higher in HCC group than in 3‐HCC group (Figure 3E). Additionally, the level of Ly6C was higher in HCC group than 3‐HAA group, suggested that Ly6C^hi^ macrophages transformed into Ly6C^lo^ macrophages after 3‐HAA treatment (Figure 3E). To further investigate the function of distinct subpopulations, we compared the differences in functional markers (CD39, CD44, CD172a, and Ki67) between the subpopulations, with the results demonstrating the obvious functional heterogeneity of proliferation and activation in different TAMCs subpopulations (Figure S3, Supporting Information).

**Figure 4:**
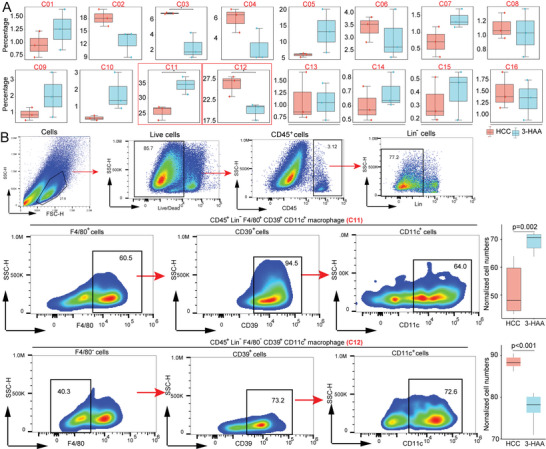
3‐HAA remodels macrophage phenotypes in the mice model of HCC. A) Bar plots of the percentage of macrophage subsets in the two groups. B) Representative flow cytometric dot plots and percentages of CD45^+^F4/80^+^CD64^+^CD39^+^CD44^+^CD11b^+^CD11c^+^PD^−^L1^+^ macrophage and CD45^+^CD64^+^CD39^+^CD44^+^CD11b^+^CD11c^+^ macrophage. *P < 0.05; **P < 0.01; ***P < 0.001. HCC, hepatocellular carcinoma; 3‐HAA, 3‐hydroxyanthranilic acid.

In addition, when the percentages of subpopulations in the two groups were compared, we identified two significantly different macrophage subpopulations: CD45^+^F4/80^+^CD64^+^CD39^+^CD44^+^CD11b^+^CD11c^+^PD‐L1^+^ macrophages (C11); and CD45^+^F4/80^−^CD64^+^CD39^+^CD44^+^CD11b^+^CD11c^+^ macrophages (C12) (**Figure** **4**A). We performed a flow cytometric analysis to validate the CyTOF results. 3‐HAA significantly increased the percentage of CD45^+^F4/80^+^CD64^+^CD39^+^CD44^+^CD11b^+^CD11c^+^PD‐L1^+^ macrophages, while decreasing the percentage of CD45^+^CD64^+^CD39^+^CD44^+^CD11b^+^CD11c^+^ macrophages (Figure 4B). Combined with figure 3A, we found that C11 was an F4/80^hi^CX3CR1^lo^Ki67^lo^MHCII^hi^ macrophages subgroup which may be derived from monocyte differentiation and had certain inflammatory chemotaxis and proliferation functions. C12 was an F4/80^lo^CD64^+^PD‐L1^lo^macrophage subgroup without the expression of inflammatory marker, this subgroup may belong to the immunosuppressive TAMCs. These findings indicate that 3‐HAA's antitumor effects may be mediated by regulating the number and function of different macrophage subtypes.

### 2.5 scRNA‐Seq Analysis Reveals Different Cell Subgroups in HCC

**Figure 5:**
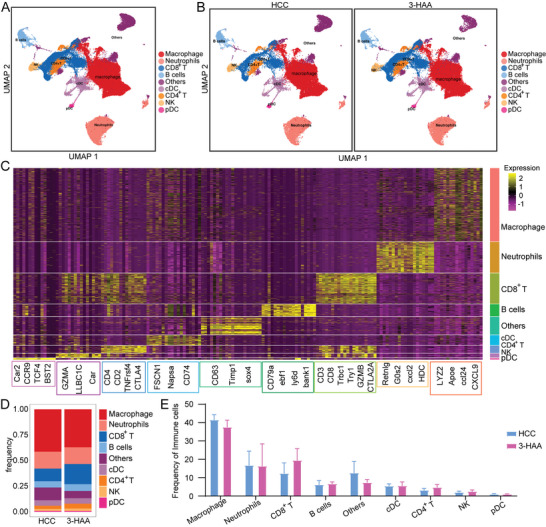
scRNA‐seq showing the nine major clusters on the immune cell island. A and B) UMAP plot of all groups and each indicated group of the nine major clusters. C) Heatmap showing the expression level of canonical genes among nine major clusters. D) The proportional distribution of the nine immune cells clusters. E) Bar plots of frequency for all types of immune cells. HCC, hepatocellular carcinoma; 3‐HAA, 3‐hydroxyanthranilic acid; UMAP, uniform manifold approximation and projection.

To further understand the tumoral immune cell remodeling mediated by 3‐HAA, we collected fresh tumor tissues from 3‐HAA group and HCC group for scRNA‐seq with the 10x drop‐seq platform (10x genomics). **Figure** **5**A showed the annotation and color codes for immune cell in HCC tissues through uniform manifold approximation and projection (UMAP). Based on the expression of marker genes, the regions can be broadly attributed to macrophage, neutrophils, CD8+ T cells, B cells, cDCs, CD4+ T cells, NK cells, and pDCs. In addition, there were several distinct spatial regions of UAMP in the 3‐HAA group and HCC group (Figure 5B). Furthermore, we defined a gene expression signature for each cell type, highlighting key markers (Figure 5C). CXCL9 plays an important role in macrophage differentiation and migration. The high expression of CXCL9 in macrophages may provide a theoretical basis for the increased proportion of macrophages in the 3‐HAA group. Besides, the high expression of LYZ2, APOE, and CCL24 on macrophages may be the key regulatory molecules in the inhibition of 3‐HAA on HCC, which is worthy of further experimental verification. However, the proportion of macrophages, neutrophils, DCs, and NK cells did not differ significantly between the 3‐HAA group and HCC group. (Figure 5D,E).

### 2.6 Heterogeneity of Tumor‐Infiltrating Myeloid Cells Defined by scRNA‐seq

**Figure 6:**
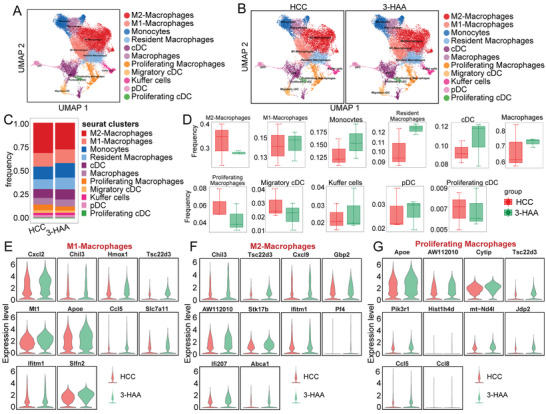
Heterogeneity in tumor‐infiltrating myeloid cells as defined by scRNA‐seq. A and B) UMAP plot of all groups and each indicated group of the major myeloid cell subpopulations. C) The proportional distribution of myeloid populations. D) Bar plots of frequency for myeloid populations. E‐G) The difference between M1, M2, and proliferating macrophages in especially expressed markers. HCC, hepatocellular carcinoma; 3‐HAA, 3‐hydroxyanthranilic acid; UMAP, uniform manifold approximation and projection.

To investigate the potential role of 3‐HAA in regulating myeloid cells in HCC, we performed scRNA‐seq on tumor tissues of 3‐HAA group and HCC group. Various myeloid subsets were identified, including kupffer cells, proliferating macrophages, macrophages, M1 macrophages, M2 macrophages, cDCs, migratory cDCs, monocytes, pDCs, proliferating cDCs, and resident macrophages (**Figure** **6**A,B; Table S3, Supporting Information). Furthermore, we observed that M2 macrophages and proliferating macrophages decreased in the 3‐HAA group compared to the HCC group (Figure 6C,D). To investigate the function of the major macrophage subtypes, we explored the unique markers expressed in M1, M2, and proliferating macrophages between the two groups. After 3‐HAA treatment, cytokine genes (*CXCL2* and *CCL5*), antioxidant gene (*Hmox1*), and immunoregulatory genes (*Chil3*) were upregulated in M1 macrophages (Figure 6E), indicted that M1 showed stronger chemotactic function in the high expression of 3‐HAA group. Interferon‐induced genes *IFI207* and cell cycle gene *Stk17b* were upregulated in M2 macrophages (Figure 6F), and the mRNA expression levels of *Apoe, AW112010, Cytip, Tsc22d3, Pik3r1, mt‐Nd4l, Jdp2*, and *CCL5*, were increased in proliferating macrophages (Figure 6G).

### 2.7 3‐HAA Regulates the Production of Inflammatory Cytokines in HCC

**Figure 7:**
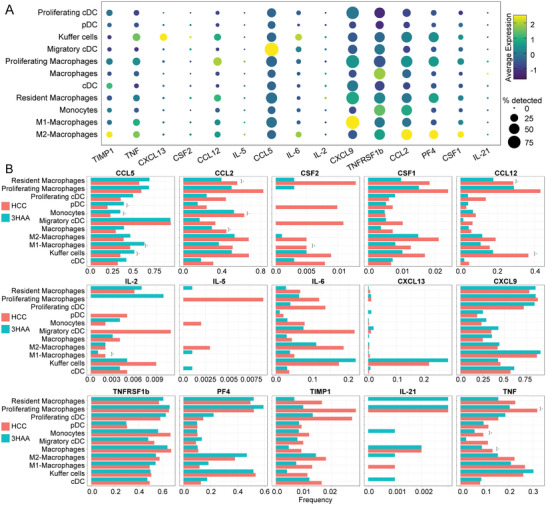
3‐HAA regulates the production of inflammatory cytokines in HCC. A) Dot plot showing the gene expression level of cytokines in each myeloid cell subpopulation. The size of the point indicates the proportion of cells that highly express a gene in this cell cluster. The color of the point indicates the level of expression of a gene in this cell cluster. B) scRNA‐seq profiling reveals that 3‐HAA alters the production of cytokines and chemokines in various cell subsets. *P < 0.05; **P < 0.01. HCC, hepatocellular carcinoma; 3‐HAA, 3‐hydroxyanthranilic acid.

Based on the research above, the plasma levels of several cytokines were found to be regulated by 3‐HAA (Figure 1D). Furthermore, we investigated the gene expression of canonical cytokines in each myeloid cell subpopulation to explore which immune cells drive these cytokines. The result indicated that IL‐6 expression was mainly restricted to kupffer cells and M2‐macrophages, whereas TNF and TNFRSF1b production was abundant in kupffer cells and macrophages. CCL5 was highly expressed in migratory cDCs. M2 macrophages were the dominant source of CCL2 (**Figure** **7**A). CSF‐1, also known as macrophage colony‐stimulating factor, which stimulates tumor growth in tissues and induces angiogenesis, was significantly upregulated in M2 macrophages (Figure 7A).

At the single‐cell level, multiple‐cell subsets, such as resident macrophages, proliferating macrophages, and pDCs, displayed significant inhibition of the proinflammatory factors TNF and IL‐6 by 3‐HAA (Figure 7B). CXCL13 was enriched in kupffer cells and upregulated in the 3‐HAA group. 3‐HAA inhibited CSF1 and CSF2 in numerous cell subsets, including resident macrophages, M2 macrophages, M1 macrophages, and kupffer cells (Figure 7B). All these findings indicate that 3‐HAA may be involved in remodeling the tumor immune microenvironment by regulating a variety of cytokines secreted by macrophages and DCs, thereby affecting the progression of HCC.

## 3 Discussion

The Trp/Kyn pathway is a major catabolic pathway for Trp metabolism and is involved in tumor progression.^[^ ^14^, ^15^ ^]^ Here, LC–MS analysis revealed that 3‐HAA, a metabolite of the Kyn metabolic pathway, was lower in HCC tissues than in adjacent nontumor tissues. The receiver operating characteristic curve analysis revealed that 3‐HAA has a high potential for use as a biomarker in the diagnosis of HCC. Although the precise role of 3‐HAA in cancer is unknown, growing evidence suggests that the kynurenine metabolite 3‐HAA has profound immunomodulatory effects on various immune cells. Fallarino et al. demonstrated that 3‐HAA activated caspase‐8 and induced Th1 cell apoptosis in vitro without requiring Fas/Fas‐L interactions.^[^ ^16^ ^]^ Similarly, Terness et al. demonstrated that 3‐HAA inhibited T cell proliferation and activation in vitro.^[^ ^17^ ^]^ In addition, 3‐HAA significantly suppressed the antigen‐independent activation of CD8+ T cells induced by antigenic stimulation. Gargaro et al. demonstrated that 3‐HAA significantly activated Foxp3+ regulatory T cells to secrete immunosuppressive transforming growth factor *β* and regulated the immunoregulatory effect of conventional dendritic cells.^[^ ^18^ ^]^ Furthermore, Kyoungran et al. demonstrated that 3‐HAA treatment modulated macrophage polarization and significantly decreased the production of pro‐inflammatory mediators by inhibiting mTOR and NF‐*κ*B activity.^[^ ^10^ ^]^ Karayama et al. reported that patients who achieved effective responses had a lower level of 3‐HAA than those who did not, and the level of 3‐HAA was effective in predicting ICI therapy in nonsmall cell lung cancer.^[^ ^19^ ^]^ Here, we found that the tumor weights and volume was smaller in the 3‐HAA group compared to the HCC group. The CyTOF results revealed that the proportion of CD45^+^F4/80^+^CD64^+^CD39^+^CD44^+^CD11b^+^CD11c^+^PD‐L1^+^ macrophages was significantly increased, while the proportion of CD45^+^F4/80^−^CD64^+^CD39^+^CD44^+^CD11b^+^CD11c^+^ macrophages was significantly decreased after 3‐HAA treatment in the mice model of HCC. The results of CyTOF were validated using flow cytometry. Our results may provide a new avenue for combating HCC progression with the 3‐HAA treatment.

We observed that the plasma levels of cytokines such as IL‐6, IL‐2, TNF, IL‐5, IL‐21, and MCP‐1 are significantly affected by 3‐HAA treatment. Strong evidence indicates that IL‐2 is involved in mediating T cell proliferation, survival, and differentiation to promote antitumor immunity.^[^ ^20^, ^21^ ^]^ Antagonists of major inflammatory cytokines can be used safely in patients with advanced‐stage cancer and may contribute to disease stabilization.^[^ ^22^ ^]^ Our findings provide a blueprint of various cytokines in the tumor microenvironment based on 3‐HAA treatment, implying that 3‐HAA treatment combined with harnessing cytokines may be an optimal setting for cytokine‐based therapy.

The phenotypic switching of Ly6C^high^ macrophages into Ly6C^low^ macrophages differentiated from bone marrow‐derived monocytes plays essential roles in tissue homeostasis and disease.^[^ ^23^, ^24^ ^]^ Numerous studies have confirmed that Ly6C^high^ macrophages create an inflammatory microenvironment that promotes tumor initiation, progression, and immune evasion, and that an increase in Ly6C^high^ monocyte recruitment predicts a poor prognosis in many types of cancer.^[^ ^25^, ^26^, ^27^ ^]^ Blocking CCL2/CCR2 signaling and Ly6C^high^ recruitment can restrain tumor metastasis in breast cancer.^[^ ^28^ ^]^ The special cell subset Ly6C^+^MHCII^−^CD11b^+^CD64^+^CD172a^+^CD44^+^ macrophage (C3/C4) was found to be upregulated after 3‐HCC treatment. Notably, 3‐HAA treatment significantly upregulated the expression levels of Ly6C in macrophage and downregulated the expression level of chemotactic CCL2 produced by macrophages in HCC tissues, suggesting that 3‐HAA may inhibit HCC progression by remodeling the immunophenotyping of macrophage by inhibiting the CCL2/CCR2 pathway.

Conclusion, our study reveals the influence and impact of 3‐HAA in regulation immune microenvironment in HCC and initially suggests 3‐HAA as a novel strategy of therapy for restoring immune function in HCC. However, the molecular mechanism of 3‐HAA in immunoregulation and the clinical data to support the therapeutic effect of 3‐HAA need further study.

## 4 Experimental Section

### Clinical Sample Collection and Preparation

The study was approved by The First Affiliated Hospital, Zhejiang University School of Medicine, and all participants signed written informed consent (project number IIT20210168B‐R1). Tumor tissues and adjacent nontumor tissues (*n* = 15) from HCC patients were obtained during surgical resection. Once the tissues were obtained during surgery, they were quickly frozen in liquid nitrogen and stored at −80 °C for further analysis.

### LC‐MS Analysis

LC–MS was performed on 15 HCC tissues and 15 adjacent nontumor tissues from 15 HCC patients, as previously described.^[^ ^29^ ^]^ In an EP tube containing ≈100 mg sample, 1 mL tissue extract, and 3 steel beads were added. The samples were then placed in a tissue grinder and ground at 55 Hz for 2 min, followed by 30 min of ultrasound. Each sample was centrifuged at 4 °C for 10 min at 12 000 rpm, and 200 µL of the supernatant was transferred to another 1.5 mL centrifuge tube. Following that, the samples were vacuum‐dried. The samples were prepared for LC–MS by dissolving them in 200 µL of 2‐chlorobenzalanine (4 ppm) solution in 50% acetonitrile and filtering the supernatant through a 0.22 µm membrane. Chromatographic separation was carried out in a Thermo Scientific Vanquish system equipped with Waters Corp ACQUITY UPLC HSS T3 (1.8 µm, 150 mm × 2.1 mm) column kept at 40 °C. The ESI‐MSn experiments were carried out on the Thermo Scientific Q Exactive HF‐X mass spectrometer with a spray voltage of 3.5 kV and −2.5 kV in positive and negative modes.

### Orthotopic Liver Cancer Model in Mice

The liver tumor cell line Hepa1‐6 was obtained from ATCC and maintained in Dulbecco's Modified Eagle's Medium (Sigma‐Aldrich, USA) supplemented with 10% fetal bovine serum (Sigma‐Aldrich, USA) in a humidified atmosphere containing 5% CO_2_ at 37 °C, as previously described.^[^ ^30^, ^31^ ^]^ Six‐week WT C57BL/6J male mice were anesthetized with 1% pentobarbital solution (Sigma–Aldrich, USA) at a dose of 50 mg kg^−1^. The mice were secured in the supine position on operating table, and their abdomen was sterilized. Sterilized tweezers and ophthalmic scissors were used to open the mice abdominal cavity. This procedure was carefully performed to avoid any cuts in the blood vessels and unnecessary bleeding. The left lobe of the liver was plucked out using a sterile cotton swab. 3 × 10^5^ Hepa1‐6 cells suspended in 10 µl Corning Matrigel (Matrigel:PBS = 1:4) were injected into the left lobe of the liver. Following the application of sterile cotton swabs to stop the bleeding, the mouse's abdomen was sutured. Finally, mice naturally woke up in the incubator. 3‐HAA was purchased from Aladdin Bio‐Chem Technology Co., Ltd. (Shanghai, China) and administered intraperitoneal injection at a dose of 100 mg/kg once daily for consecutive 14 days. This experiment was approved by the Animal Experimental Ethics Committee of The First Affiliated Hospital, Zhejiang University School of Medicine (project number 20221072).

### Quantitative Analysis of Cytokines

A total of 40 cytokines in mouse plasma samples (3‐HAA group versus HCC group, *n* = 10 vs *n* = 10) were tested using Quantibody Mouse Inflammation Array Kit (RayBiotech, USA) according to the manufacturer's instructions. The information regarding these 40 cytokines is presented in Table S4, Supporting Information. The protocol is described in the previous studies.^[^ ^32^, ^33^ ^]^ The concentrations of various cytokines were analyzed using the Student's *t*‐test.

### CyTOF Analysis

Six fresh HCC tissues (3‐HAA group versus HCC group, *n* = 3 vs *n* = 3) removed surgically from mice models were stored and transported at a low temperature. Tissues were digested using the Tumor Dissociation Kit (Miltenyi Biotec, Germany, Order #130‐105‐807), and then cells were filtered, washed, and stained. A total of 42 metal‐isotope‐tagged antibodies were used in the study. A final concentration of 0.25 µM 194Pt (1 mM) dead and live staining solution were prepared. Cells were resuspended in 100 µL of 194Pt dead and live staining solution and stained on ice for 5 min (100 µL per sample). 1 mL of FACS buffer (1.25% BSA in PBS, BD Bioscience, USA) was added to each sample and centrifuged at 400 g at 4 °C for 5 min. After discarding the supernatant, 50 µL of Block mix was added to each sample, the cells were resuspended, and the samples were blocked on ice for 20 min. Following incubation, the antibody mix was added directly. The cells were stained for 30 min on ice. The Fix and Perm Buffers were used to prepare a staining solution with a final concentration of 250 n. 200 µL of resuspended cells were taken from each sample and stained for 1 h at 24 °C. All CyTOF data were normalized and manually gated in FlowJo (version 10). Circle the single and intact live cells, and label the CD45^+^ immune cells. The x‐shift algorithm was used for cell subgroup clustering, annotation, t‐SNE dimensionality reduction visualization, and statistical analysis.^[^ ^34^ ^]^

### Flow Cytometry

Six disaggregated mice model tumor tissues (3‐HAA group versus HCC group, *n* = 6 vs *n* = 6) infiltrating immune cells were incubated with anti‐mouse antibodies CD45‐FITC, LIN‐(CD3‐PE, CD19‐PE, CD49B‐PE, GR‐1‐PE, SiglecF‐PE), F4/80‐BV421, CD11c‐PE‐CY7, CD39‐BV711, L/D‐7AAD (BD Biosciences, USA). All the antibodies were purchased from BD Biosciences. A Fortessa cell analyzer (BD Bioscience, USA) was used to test stained cells.

### scRNA‐Seq Analysis

scRNA‐seq was performed on tumor tissues (3‐HAA group versus HCC group, *n* = 3 vs *n* = 3) from mice model of HCC.^[^ ^35^, ^36^ ^]^ First, the tissues were processed into single‐cell suspensions for cell counting and cell viability evaluation. The prepared single‐cell suspension was combined with gel beads containing barcode information and a mixture of enzymes. Next, cell lysis and reverse transcription reactions were carried out in the Gel Beads‐in emulsions (GEMs). The 10x Genomics barcodes were linked to the cDNA products in the corresponding cells during this process. The GEMs were then disrupted, and the cDNAs from different cells were mixed. Based on this, PCR amplification and quality inspection (amplified fragment size and yield of amplified product) were performed on the 50 000‐100 000 reads measured per cell using the Illumina Hiseq PE150 sequencing strategy (Illumina, USA).

Low‐quality reads, adapter contamination, and a high N content of unknown bases were removed from sequencing raw data. Cell Ranger was used to performing alignment with the reference genome, demultiplexing, determining the effective cell number, and constructing a gene‐barcode expression profile matrix.^[^ ^37^ ^]^ Based on the gene‐barcode expression profile, the Seurat R package was used for expression profile preprocessing and cluster analysis.^[^ ^38^ ^]^ UMAP was used to reduce dimensionality and display the obtained subgroups graphically, and a characteristic marker genes analysis was performed on the subgroups.^[^ ^39^ ^]^

### Statistical Analysis

The statistical analyses were carried out using SPSS 25.0 and R 3.6.1 software. Statistical differences of a continuous variable between the two groups were analyzed by unpaired Student's *t*‐test. The data are presented as mean ± SD. FACS analyses were performed using FlowJo 10.6.2 (BD Biosciences, USA). *P* < 0.05 (two‐sided) was considered statistically significant.

## Conflict of Interest

The authors declare no conflict of interest.

## Author Contributions

C.X., X.G., Q.Z., and Q.S. contributed equally to this work. L.L. and J.L. designed and guided the study. C.X., X.G., Q.Z., and Q.S. participated in the overall experiments and wrote the draft. X.Y., Q.C., J.J., Y.S., and Z.B. collected and analyzed data. All authors read and approved the final manuscript.

## Supporting information

Supporting information

## Data Availability

The data that support the findings of this study are available from the corresponding author upon reasonable request. The original data of scRNA‐seq can be accessed in GEO DataSet (GSE226510).
