# Supplementary material for: Effects of 3‐HAA on HCC by Regulating the Heterogeneous Macrophages—A scRNA‐Seq Analysis
Source: Adv Sci (Weinh). 2023 Apr 4;10(16):2207074. doi: 10.1002/advs.202207074 (PMC10238176; doi:10.1002/advs.202207074)
Supplement: Supplementary file 1 — Supporting information [file ADVS-10-2207074-s001.pdf]

ADVANCED SCIENCE
Open Access

Supporting Information

for *Adv. Sci.*, DOI 10.1002/advs.202207074

Effects of 3-HAA on HCC by Regulating the Heterogeneous Macrophages—A scRNA-Seq Analysis

Chen Xue, Xinyu Gu, Qiuxian Zheng, Qingmiao Shi, Xin Yuan, Qingfei Chu, Junjun Jia, Yuanshuai Su, Zhengyi Bao, Juan Lu and Lanjuan Li**

Supporting information

Effects of 3-HAA on HCC by regulating the heterogeneous macrophages—A scRNA-seq analysis

Chen Xue[1†], Xinyu Gu[1†], Qiuxian Zheng[1†], Qingmiao Shi[1†], Xin Yuan[1], Qingfei Chu[1], Junjun Jia[2], Yuanshuai Su[1], Zhengyi Bao[1], Juan Lu[1*], Lanjuan Li[1*]

[1] State Key Laboratory for Diagnosis and Treatment of Infectious Diseases, National Clinical Research Center for Infectious Diseases, National Medical Center for Infectious Diseases, Collaborative Innovation Center for Diagnosis and Treatment of Infectious Diseases, The First Affiliated Hospital, Zhejiang University School of Medicine, Hangzhou, 310003, China.

[2] Division of Hepatobiliary and Pancreatic Surgery, Department of Surgery, The First Affiliated Hospital, Zhejiang University School of Medicine, Hangzhou, 310003, China.

[†]These authors contributed equally

***Corresponding author: Lanjuan Li & Juan Lu**

State Key Laboratory for Diagnosis and Treatment of Infectious Diseases, The First Affiliated Hospital, Zhejiang University School of Medicine, No. 79 Qingchun Road, Shangcheng District, Hangzhou, Zhejiang 310003, China. Tel: +86-0571-87236459; Email: ljli@zju.edu.cn (Lanjuan Li), lujuanzju@zju.edu.cn (Juan Lu).

Supplementary figures and tables

1. Supplementary figures S1-3

Supplementary figure S1 3

Supplementary figure S2 4

Supplementary figure S3 5

2. Supplementary tables S1-4

Supplementary table S1 6

Supplementary table S2 8

Supplementary table S3 9

Supplementary table S4 10

Supplementary Figures

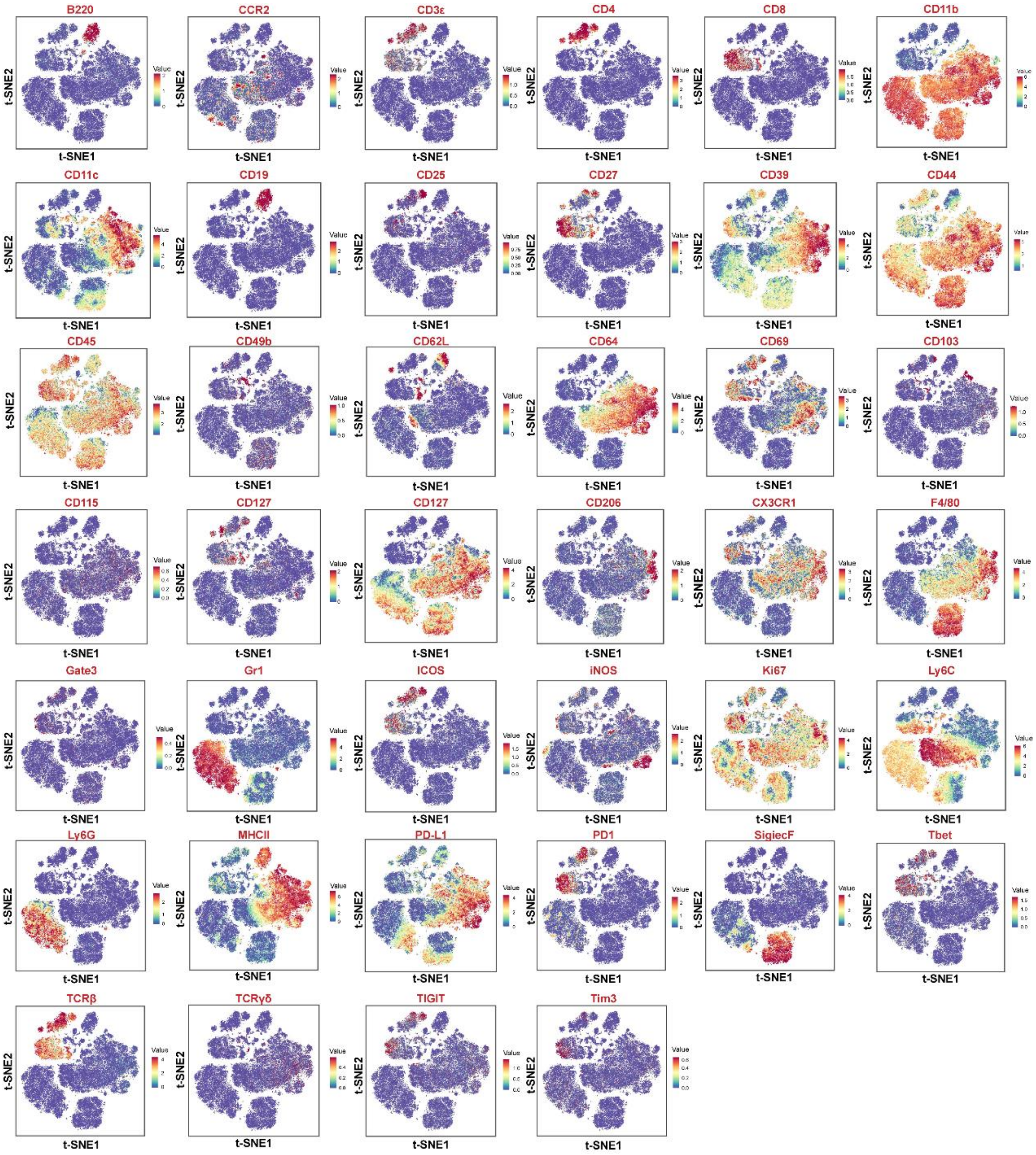

Figure S1. t-SNE maps of 40 selected panel markers.

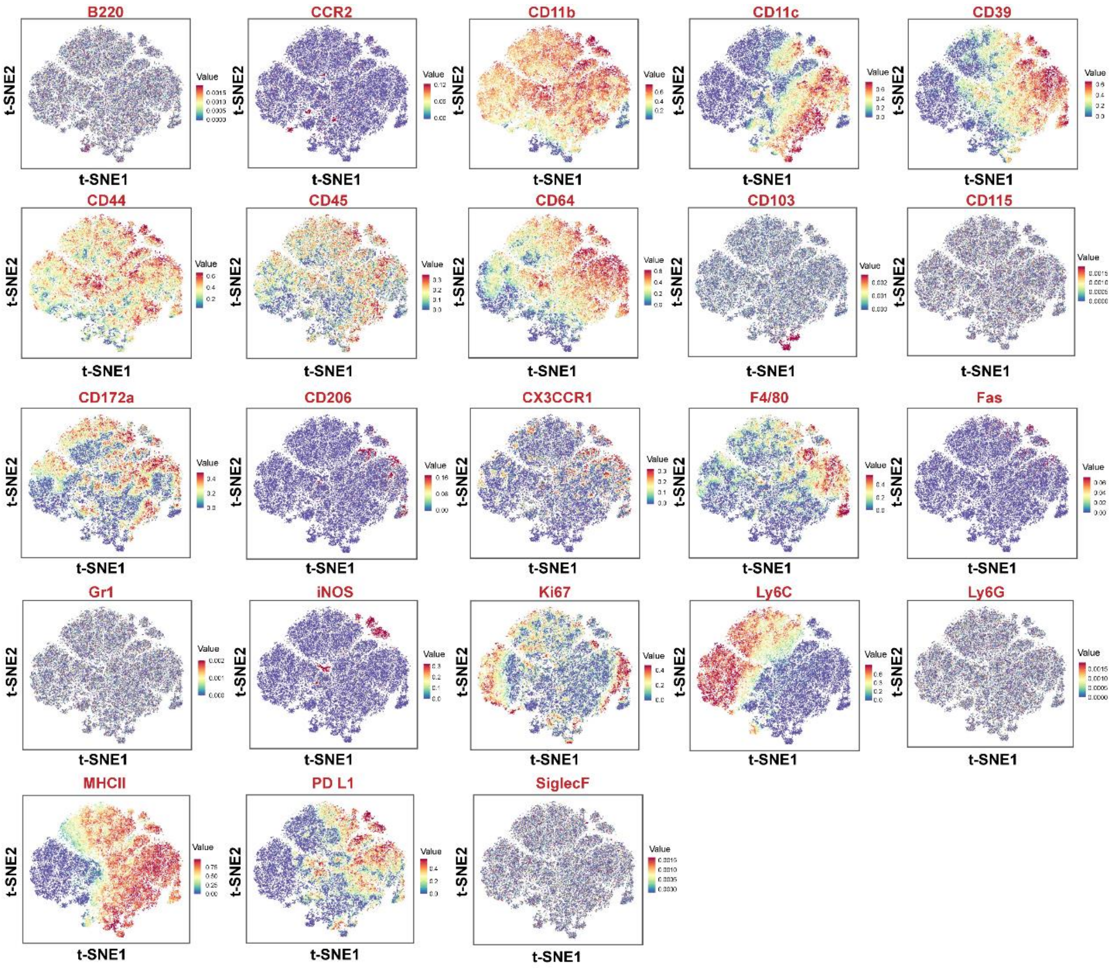

Figure S2. t-SNE maps of 23 special markers representing myeloid populations.

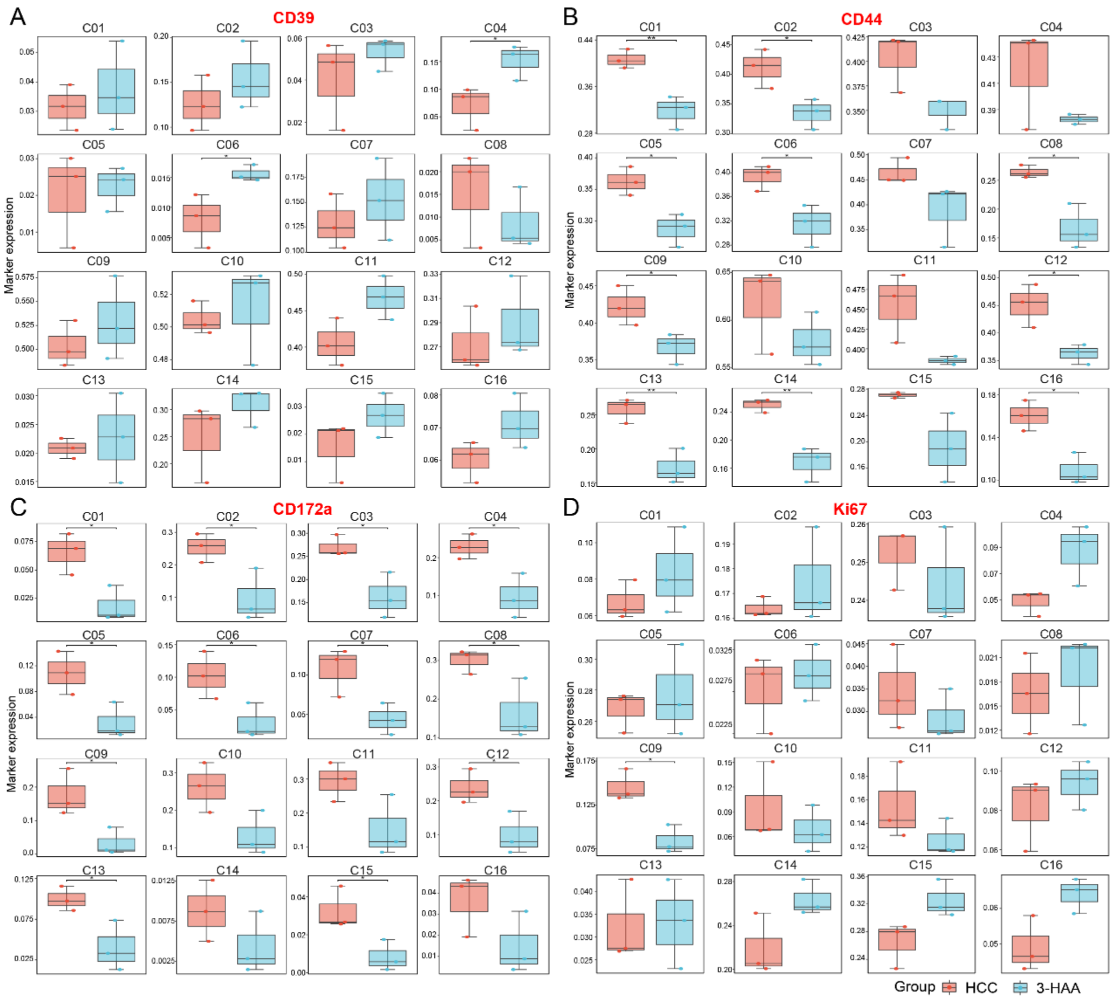

Figure S3. Bar plots of differently expressed functional markers in myeloid populations.

Table S1. The details of all immune cell clusters, cell types, and subtypes were analyzed by CyTOF

Cell types	Clusters	Marker expression
$CD4^{+}TN$	C01	$CD3e^{+}TCR\beta^{+}CD27^{+}CD62L^{+}CD127^{+}CD4^{+}$
$CD8^{+}TN$	C02	$CD103^{+}TCR\beta^{+}CD27^{+}CD62L^{+}CD127^{+}CD8^{+}$
$CD4^{+}TE$	C03	$TCR\beta^{+}CD127^{+}CD4^{+}$
$CD4^{+}Treg$	C04	$CD3e^{+}ICOS^{+}Ki67^{+}CD39^{+}TIGIT^{+}TCR\beta^{+}CD69^{+}CD25^{+}CD27^{+}MHCII^{+}CD4^{+}$
$CD4^{+}TE$	C05	$CD3e^{+}PD1^{+}CX3CR1^{+}ICOS^{+}Ki67^{+}CD39^{+}TIGIT^{+}TCR\beta^{+}CD69^{+}CD27^{+}MHCII^{+}CD4^{+}$
$CD4^{+}TE$	C06	$PD1^{+}ICOS^{+}CD39^{+}TCR\beta^{+}CD69^{+}CD4^{+}$
B cells	C07	$CD19^{+}B220^{+}CD62L^{+}MHCII^{+}$
B cells	C08	$CD19^{+}B220^{+}MHCII^{+}$
B cells	C09	$CD19^{+}B220^{+}MHCII^{+}$
DC	C10	$Ki67^{+}CD103^{+}CD39^{+}CD11c^{+}PD\text{-}L1^{+}MHCII^{+}$
B cells	C11	$Ly6C^{+}Fas^{+}Ki67^{+}CD39^{+}TIGIT^{+}CD69^{+}PD\text{-}L1^{+}MHCII^{+}CD11b^{+}$
Macrophage	C12	$CX3CR1^{+}CD39^{+}F4/80^{+}CD64^{+}CD206^{+}PD\text{-}L1^{+}MHCII^{+}CD11b^{+}$
Macrophage	C13	$Fas^{+}CX3CR1^{+}CD39^{+}F4/80^{+}iNOS^{+}CD11c^{+}CD64^{+}PD\text{-}L1^{+}CD172a^{+}MHCII^{+}CD11b^{+}$
Macrophage	C14	$Ly6C^{+}CX3CR1^{+}Ki67^{+}CD39^{+}F4/80^{+}CD69^{+}CD11c^{+}CD64^{+}PD\text{-}L1^{+}CD172a^{+}MHCII^{+}CD11b^{+}$
NK	C15	$CD49b^{+}Ki67^{+}PD\text{-}L1^{+}CD11b^{+}$

Cell types	Clusters	Marker expression
NK	C16	$CD49b^{+}CD69^{+}CD11c^{+}CD27^{+}CD62L^{+}CD11b^{+}$
Others	C17	$Ly6C^{+}Ki67^{+}PD\text{-}L1^{+}$
Neutrophils	C18	$Ly6C^{+}Gr1^{+}Ly6G^{+}Ki67^{+}CD39^{+}CD172a^{+}CD11b^{+}$
Eosinophils	C19	$Ly6C^{+}Ki67^{+}CD39^{+}F4/80^{+}CD172a^{+}SiglecF^{+}CD11b^{+}$
Eosinophils	C20	$CD39^{+}F4/80^{+}CD11c^{+}PD\text{-}L1^{+}CD172a^{+}SiglecF^{+}CD11b^{+}$
Eosinophils	C21	$Ly6C^{+}CD39^{+}F4/80^{+}CD11c^{+}PD\text{-}L1^{+}CD172a^{+}SiglecF^{+}CD11b^{+}$
Macrophage	C22	$Ly6C^{+}CX3CR1^{+}Ki67^{+}CD39^{+}F4/80^{+}CD64^{+}CD172a^{+}MHCII^{+}CD11b^{+}$
Macrophage	C23	$Ly6C^{+}F4/80^{+}CD64^{+}CD172a^{+}CD11b^{+}$
Macrophage	C24	$Ly6C^{+}CX3CR1^{+}F4/80^{+}CD64^{+}CD172a^{+}CD11b^{+}$
NK	C25	$CD39^{+}CD69^{+}CD11c^{+}$
NK	C26	$Ly6C^{+}Tbet^{+}CX3CR1^{+}CD49b^{+}CD11c^{+}CD11b^{+}$
pDC	C27	$Ly6C^{+}CD39^{+}B220^{+}CD11c^{+}CD172a^{+}MHCII^{+}CD4^{+}$
Others	C28	$Ly6C^{+}Ki67^{+}CD11c^{+}$
γδT	C29	$Ly6C^{+}TCR\gamma\delta^{+}CD27^{+}CD62L^{+}CD127^{+}$
$CD8^{+}T$	C30	$Ly6C^{+}TCR\beta^{+}CD27^{+}CD62L^{+}CD127^{+}$
$CD8^{+}TN$	C31	$Ly6C^{+}PD1^{+}CX3CR1^{+}Ki67^{+}CD39^{+}TCR\beta^{+}CD69^{+}CD27^{+}MHCII^{+}CD8^{+}$
$CD8^{+}TN$	C32	$Ly6C^{+}CD39^{+}TCR\beta^{+}CD69^{+}CD11c^{+}$

Table S2. The details of all myeloid cell clusters, cell types, and subtypes were analyzed by CyTOF

Cell types	Subtypes	Clusters	Marker expression
macrophage		C01	$Ly6C^{+}MHCII^{+}CD11b^{+}CD44^{+}$
macrophage		C02	$Ly6C^{+}MHCII^{+}CD11b^{+}CD64^{+}CD44^{+}$
macrophage		C03	$Ly6C^{+}$MHCII-$Ki67^{+}CD11b^{+}CD64^{+}CD172a^{+}CD44^{+}$
macrophage		C04	$Ly6C^{+}$MHCII-Ki67-$CD11b^{+}CD64^{+}CD172a^{+}CD44^{+}$
macrophage		C05	$Ly6C^{+}$MHCII-$Ki67^{+}CD11b^{+}CD44^{+}$
macrophage		C06	$Ly6C^{+}$MHCII-$CD11b^{+}CD44^{+}$
macrophage		C07	$CD11b^{+}CD64^{+}CD44^{+}$
macrophage		C08	$CD11c^{+}CD11b^{+}CD172a^{+}$
macrophage	M2	C09	$F4/80^{+}CD64^{+}CD39^{+}CD44^{+}$
macrophage	M1	C10	$F4/80^{+}CD64^{+}CD39^{+}CD44^{+}CD11b^{+}iNOS^{+}PD\text{-}L1^{+}$
macrophage		C11	$F4/80^{+}CD64^{+}CD39^{+}CD44^{+}CD11b^{+}CD11c^{+}PD\text{-}L1^{+}$
macrophage		C12	$CD64^{+}CD39^{+}CD44^{+}CD11b^{+}CD11c^{+}$
pDC		C13	$CD11c^{+}MHCII^{+}Ly6C^{+}$
cDC		C14	$CD11c^{+}MHCII^{+}Ki67^{+}CD39^{+}CD103^{+}$
cDC		C15	$CD11c^{+}MHCII^{+}Ki67^{+}$
cDC		C16	$CD11c^{+}MHCII^{+}PD\text{-}L1^{+}$

Table S3. The details of all myeloid cell clusters, cell types, and subtypes were analyzed by scRNA-seq

Clusters	Cell types	Subtypes	Key Marker
Cluster0	M2-Macrophages	$Fn1^{+}$M2	$Arg1^{+}Cd14^{+}Lyz2^{+}$
Cluster01	M1-Macrophages	$Cxcl9^{+}$M1	$H2\text{-}Dmb1^{+}Lyz2^{+}$
Cluster02	M2-Macrophages	$Arg1^{+}$M2	$Arg1^{+}Lyz2^{+}$
Cluster03	Monocytes	$Chil3^{+}$Monocytes	$Chil3^{+}Ly6c2^{+}Lyz2^{+}$
Cluster04	Resident Macrophages	$CD81^{+}$Macrophages	$Cd81^{+}C1qa^{+}Lyz2^{+}$
Cluster05	cDC	$CD209a^{+}$DC	$Cd209a^{+}H2\text{-}Aa^{+}Clec10a^{+}$
Cluster06	Macrophages	Ace^{+}Macrophages	$Cd300a^{+}Lyz2^{+}$
Cluster07	Proliferating Macrophages	$Mki67^{+}$Macrophages	$Mki67^{+}Lyz2^{+}$
Cluster08	Migratory cDC	$Ccr7^{+}Ccl5^{+}$DC	$Ccr7^{+}Ccl5^{+}Tbc1d4^{+}Cst3^{+}$
Cluster09	Monocytes	Ace^{+}Monocytes	$Ace^{+}Ly6c2^{+}$
Cluster10	Kuffer cells	$Clec4f^{+}$Kuffer cells	$Clec4f^{+}$
Cluster11	pDC	$SiglecH^{+}$pDC	$Siglecf^{+}$
Cluster12	Proliferating Macrophages	$Mcm3^{+}$Macrophages	$Mcm3^{+}Mcm6^{+}C1qa^{+}$
Cluster13	cDC	$Xcr1^{+}$cDC	$Xcr1^{+}Cst3^{+}Clec9a^{+}$
Cluster14	M2-Macrophages	$Arg1^{+}Saa3^{+}$M2	$Arg1^{+}C1qa^{+}$
Cluster15	Proliferating cDC	$Mcm3^{+}$cDC	$Mcm3^{+}Mcm6^{+}CST3^{+}$
Cluster16	pDC	$CD209d^{+}$pDC	$Cd209d^{+}Cd209a^{+}Siglech^{+}$

Table S4. The Quantibody Mouse Inflammation Array Kit contains information of 40 cytokines

Name	Average expression		Fold change	logFC	*P*-value	Adjust *P*-value
	3-HAA group	HCC group				
TIMP-1	12.75564	10.710364	4.12752	2.045275	1.43E-11	5.73E-10
TNFa	4.377788	6.538326	0.223673	-2.16054	7.33E-11	1.47E-09
BLC	11.71714	9.799796	3.777275	1.917346	8.98E-07	1.20E-05
GM-CSF	2.810518	4.086877	0.412836	-1.27636	3.72E-05	0.000305109
MCP-5	5.612818	4.620736	1.989053	0.992081	3.81E-05	0.000305109
IL-5	5.363004	6.441348	0.473572	-1.07834	0.000116	0.000775834
RANTES	6.002099	4.510055	2.812873	1.492044	0.000259	0.001480086
IL-6	7.206499	5.501432	3.260441	1.705067	0.000511	0.002553632
Leptin	10.37657	9.113039	2.400821	1.263528	0.001701	0.007558416
IL-2	3.152177	4.078661	0.526139	-0.92648	0.00256	0.010056094
MIG	9.073243	7.526394	2.921782	1.546848	0.002765	0.010056094
TNF RII	6.544429	7.504444	0.514052	-0.96002	0.011813	0.039375828
MCP-1	5.062386	6.435346	0.386098	-1.37296	0.015084	0.046413391
PF4	11.29392	11.895787	0.6589	-0.60187	0.01782	0.050914471
MCSF	3.313862	2.305273	2.011943	1.00859	0.021437	0.057164209
IL-3	3.171101	2.189877	1.97414	0.981224	0.02646	0.066151083
IL-21	7.125611	6.458344	1.588062	0.667267	0.032672	0.076875037
MIP-1g	8.509542	8.723811	0.861983	-0.21427	0.056067	0.124593099

Name	Average expression		Fold change	logFC	*P*-value	Adjust *P*-value
	3-HAA group	HCC group				
Eotaxin-2	3.78015	3.143810	1.55438	0.63634	0.072737	0.148316922
IL-4	2.607128	3.039943	0.740815	-0.43282	0.080715	0.148316922
IL-17	3.903029	3.142582	1.694015	0.760447	0.081574	0.148316922
TARC	4.876337	4.293003	1.498308	0.583334	0.076627	0.148316922
Eotaxin	6.139995	5.851608	1.221274	0.288387	0.08905	0.148416743
KC	5.609233	5.038121	1.485668	0.571112	0.085922	0.148416743
IL-12p70	4.566027	3.846635	1.646489	0.719393	0.109385	0.175015778
IL-13	6.870799	6.282661	1.503306	0.588139	0.187424	0.288344496
G-CSF	7.973006	8.645769	0.627304	-0.67276	0.211464	0.313280535
IL-1a	2.885475	2.655678	1.17267	0.229797	0.300054	0.428648632
IL-7	8.934043	8.484258	1.365837	0.449785	0.341399	0.470895216
IL-10	5.228037	5.511335	0.821711	-0.2833	0.365852	0.487802505
CD30L	2.459745	2.074961	1.305665	0.384785	0.40115	0.517612996
Fas L	6.232679	5.596861	1.553818	0.635818	0.457481	0.570968336
IL-1b	4.044307	4.401684	0.780583	-0.35738	0.471049	0.570968336
TCA-3	3.535392	3.689248	0.898845	-0.15386	0.565541	0.665341843
LIX	10.47742	10.176661	1.231789	0.300755	0.665115	0.739863335
MIP-1a	2.936758	2.707100	1.172557	0.229658	0.665877	0.739863335
IL-15	10.84046	11.229492	0.763641	-0.38903	0.756719	0.818075035

**Name**	**Average expression**		**Fold change**	**logFC**	***P*-value**	**Adjust *P*-value**
	**3-HAA group**	**HCC group**				
ICAM-1	9.81125	9.767351	1.030897	0.0439	0.782412	0.823591896
IFNg	5.576423	5.536569	1.02801	0.039854	0.898118	0.921147128
TNF RI	7.087993	7.098878	0.992484	-0.01088	0.937442	0.937442013
